# A simple filter model to guide the allocation of healthcare resources for improving the treatment of depression among cancer patients

**DOI:** 10.1186/s12885-018-4009-2

**Published:** 2018-02-06

**Authors:** Robert W. Sanson-Fisher, Natasha E. Noble, Andrew M. Searles, Simon Deeming, Rochelle E. Smits, Christopher J. Oldmeadow, Jamie Bryant

**Affiliations:** 1 Priority Research Centre for Health Behaviour, University of Newcastle, Callaghan, NSW Australia; 2 School of Medicine and Public Health, Faculty of Health and Medicine, University of Newcastle, Callaghan, NSW Australia; 3 Hunter Medical Research Institute, New Lambton Heights, NSW Australia; 4 Centre for Clinical Epidemiology and Biostatistics, University of Newcastle, Callaghan, NSW Australia

**Keywords:** Depression, Cancer, Oncology, Modelling, Costs, Patient outcomes, Decision aid, Filter

## Abstract

**Background:**

Depression is highly prevalent yet often poorly detected and treated among cancer patients. In light of the move towards evidence-based healthcare policy, we have developed a simple tool that can assist policy makers, organisations and researchers to logically think through the steps involved in improving patient outcomes, and to help guide decisions about where to allocate resources.

**Methods:**

The model assumes that a series of filters operate to determine outcomes and cost-effectiveness associated with depression care for cancer patients, including: detection of depression, provider response to detection, patient acceptance of treatment, and effectiveness of treatment provided. To illustrate the utility of the model, hypothetical data for baseline and four scenarios in which filter outcomes were improved by 15% were entered into the model.

**Results:**

The model provides outcomes including: number of people successfully treated, total costs per scenario, and the incremental cost-effectiveness ratio per scenario compared to baseline. The hypothetical data entered into the model illustrate the relative effectiveness (in terms of the number of additional incremental successes) and relative cost-effectiveness (in terms of cost per successful outcome and total cost) of making changes at each step or filter.

**Conclusions:**

The model provides a readily accessible tool to assist decision makers to think through the steps involved in improving depression outcomes for cancer patents. It provides transparent guidance about how to best allocate resources, and highlights areas where more reliable data are needed. The filter model presents an opportunity to improve on current practice by ensuring that a logical approach, which takes into account the available evidence, is applied to decision making.

## Background

### How can the treatment of depression among cancer patients be improved?

Depression is a significant problem for cancer patients. The rate of occurrence of major depression among cancer patients is approximately two to four times that of the general population [1]. Depressive symptoms and distress are associated with negative outcomes and disability, including more rapidly progressing cancer symptoms, more metastasis, pain, and poorer quality of life, compared with non-depressed cancer patients [2, 3]. Yet research indicates that depression and distress are under-recognised and under-treated among cancer patients [4–6]. While routine screening for distress is mandated as standard practice in cancer treatment settings [7, 8], there is only sparse evidence that such interventions are of benefit to patients [9]. Why is this so? Clearly, screening needs to be linked to other changes in the system of care to increase the provision of effective treatment [1, 10, 11]. Other factors which affect the provision of treatment include whether providers refer or offer treatment services to depressed patients, and whether patients accept an offer of treatment [12, 13]. If depression outcomes for cancer patients are to be improved, the range of relevant steps and influences on outcomes need to be adequately considered.

### How should decisions about allocating resources to improve patient outcomes be made?

Following the move towards evidence-based medicine, evidence-based policy is also being encouraged in all areas of public service, including health care [14]. However, reviews of public health sector decisions suggest that research currently has little direct influence on decision making [14, 15]. Policymakers tend to rely on other types of evidence, such as personal experience, or the opinions of eminent colleagues, rather than research findings [14].

A range of methods are available to assist policy makers and organisations to make evidence-based decisions about the allocation of healthcare resources. For example, decision analytic modelling is a systematic process which utilises the best available information to inform a decision when faced with various sources of uncertainty [16, 17]. Other decision making tools include techniques such as cost-effectiveness and cost benefit analysis. However, such techniques are often highly complex and require advanced skills to implement, as well as a significant investment of time and resources [18, 19]. Given the need to move towards evidence-based healthcare policy, and the limitations of utilising the currently available decision tools, there is a need for a simple tool that can assist policy makers, organisations and researchers to logically think through the steps involved in improving patient outcomes, and to make the best use of the available data. Such a tool will also serve to highlight where additional data are needed to support evidence-based decision making.

### A simple “filter model” to guide decisions about the investment of resources to improve the treatment of depression among cancer patients

In light of the constraints mentioned above, we have developed a simple ‘filter model’ to assist decision and policy makers think through some of the key steps that influence patient outcomes in depression care in cancer. The model will help guide decisions about where to best allocate resources to improve outcomes based on available evidence. The filter model combines epidemiological, statistical and economic approaches to guide policy decision making, and aims to increase the transparency of the decision making process by identifying the factors that contribute to the decision. The filter model forms a checklist of important considerations along the path of policy development, and serves to highlight those steps or aspects of care where research evidence is lacking.

In this paper we describe the application of the filter model to the allocation of resources in the treatment of depression for cancer patients. The model assumes that a series of filters operate to determine outcomes and cost-effectiveness associated with depression care. The model allows for data and costs to be entered at each step, and provides a range of metrics which allow outcome scenarios to be compared to a baseline. Although the model is set up to explore the treatment of depression in cancer patients, it can also potentially be applied to similar policy and healthcare resource allocation decisions, such as the treatment of obesity, or provision of smoking cessation strategies in General Practice.

### Aims

The aims of this paper were to: a) illustrate some of the key steps which operate to determine depression outcomes for cancer patients; b) provide decision and policy makers with a simple tool for guiding decisions about how to allocate resources to improve patient outcomes; and c) highlight areas of the literature where more research about depression care for cancer patients is needed. The filter model is a currently a theoretical tool which can be empirically tested to explore its utility and reliability.

## Method

### Definition of the model filters

**Fig. 1:**
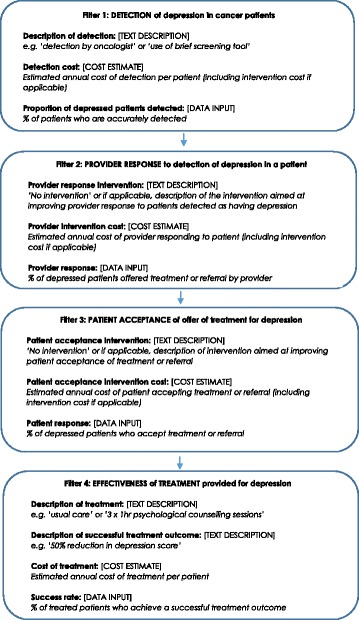
Key filters included in the filter model for allocation of healthcare resources in improving treatment of depression in cancer

Four key filters were included in the model as outlined in Fig. 1. These filters included: a) Detection of depression; b) Provider response to detection; c) Patient acceptance of treatment for depression; and d) Effectiveness of the treatment offered for depression. While there are a range of additional filters which might also influence depression outcomes among cancer patients, these four were drawn from the literature as representing those factors likely to have the greatest influence on patient outcomes.

Numerous authors note the poor levels of detection of depression by providers [4–6, 10, 12], and the role that screening can play in improving detection [12, 20]. Estimates of the correct rate of detection of depression among cancer patients by clinician judgement alone range from 5 to 37% [4, 21, 22], while the use of screening tools has been shown to improve the recognition of depression [23]. Similarly, a large body of research has focussed on the effectiveness of treatments for depression, including psychological and pharmacological approaches [24, 25], and more recently, collaborative care models [26]. Collaborative care approaches have demonstrated significant treatment success [27–29].

However, the receipt of care following detection is a key limiting factor [7, 11]. Screening for depression is unlikely to benefit patients unless it is accompanied by strategies such as providing clinicians with an interpretation of scores, mandating follow-up, and training or other clinician support [11]. In a review of barriers to the treatment of depression in cancer care, Greenberg 2004 reported the lack of provider referral and lack of patient awareness of treatment services as major barriers to the receipt of care [12]. Mitchell 2013 also reported patient lack of acceptance of treatment offered for depression as a key barrier to the receipt of care [13]. This is illustrated by reports that suggest fewer than 10% of cancer patients with significant distress are referred for psychosocial care [30]. Other authors report that only approximately one-quarter of cancer patients with depression receive treatment [31, 32]. Similarly, across several studies, only 36% of distressed cancer patients expressed a desire for help [33], less than a quarter of lung cancer patients indicated an interest in receiving help for their distress [34], and less than a third of cancer outpatients accepted an offer of help for distress [35]. Some of the reasons patients may decline treatment include a preference to self-manage, or a perception that symptoms are not severe enough to require treatment [35].

### Model design

The filter model operates within an excel spreadsheet and uses pre-defined cell algorithms. Text descriptions and numerical data, including costs, are entered into the model for a number of background parameters (including defining the nature and size of the total population and target group) and parameters reflecting attributes of each of the four model filters:

1. Detection of depression: includes a text description of how detection is undertaken, the cost associated, and the rate of correct identification of cases of depression;
2. Provider response to detection: the proportion of cancer patients who are offered treatment or a referral for treatment in response to having been identified as having depression, and associated cost;
3. Patient acceptance of treatment: the proportion of cancer patients that would be willing to accept assistance if offered some kind of treatment for depression, and associated cost;
4. Treatment effectiveness:. The proportion of patients that are successfully treated for depression (out of those that accepted treatment), and associated cost.

The model allows the user to create multiple hypothetical intervention and usual care scenarios to compare outcomes under a range of assumptions about the input data. For example, the user could model the outcomes associated with adopting a range of different approaches to the detection of depression, such as ultra-short, short, and interview style screening tools, including the anticipated cost of each approach.

Given the input data representing the background and filter model parameters, the following outcomes are estimated for each of the scenarios of interest:

1. Cost per patient: Aggregate cost of the treatment pathway for all patients, divided by the number of patients who participate in treatment;
2. Cost per successful outcome: Aggregate cost of the treatment pathway for all patients, divided by the number of patients who are successfully treated for depression;
3. Incremental cost compared to baseline: Additional aggregate cost of the treatment pathway for all patients under each scenario compared to the baseline scenario;
4. Incremental number of successes compared to baseline: The number of additional patients who achieve a successful outcome under each scenario compared to the baseline scenario;
5. Incremental cost-effectiveness ratio (ICER): Incremental cost compared to baseline (c) divided by the incremental number of successes compared to baseline (d). The ICER is the ratio of the change in cost to the change in effectiveness of each scenario compared to the baseline. It provides an estimate of the additional cost per successful outcome under each scenario compared to baseline;

- Policy advice: The model indicates whether each scenario is more or less expensive (incremental cost) and more or less effective (incremental number of successes) compared to the baseline scenario.

### Procedure

In order to illustrate use of the model for highlighting key steps which contribute to depression outcomes for cancer patients, and as a decision tool for how resources might be allocated to improve patient outcomes, hypothetical data for baseline and four different scenarios were entered into the model. The four scenarios modelled a hypothetical 15% improvement from baseline care in each of the 4 filters: detection of depression (from 20% at baseline to 35% in scenario 1), provider response to detection of depression (from 70% at baseline to 85% in scenario 2), patient acceptance of an offer of treatment for depression (from 30% at baseline to 45% in scenario 3), and the effectiveness of treatment offered for depression (from 30% at baseline to 45% in scenario 4). Arbitrary costs associated with baseline care and with achieving these improvements were also entered into the model.

## Results

Input data used and the results of the modelling of the hypothetical scenarios are presented in Table 1.

**Table 1:** Model parameters and output under hypothetical usual care and four scenarios of improvement above baseline

Model Parameters	Baseline	Scenario 1: Increase detection	Scenario 2: Increase provider response	Scenario 3: Increase patient acceptance	Scenario 4: Increase treatment effectiveness
*Population*					
Arbitrary population of cancer patients	10,000	10,000	10,000	10,000	10,000
*Target group*					
Cancer patients with depression	15% *n* = 55,500	15% *n* = 55,500	15% *n* = 55,500	15% *n* = 55,500	15% *n* = 55,500
*Filter 1: detection*					
Detection (description)	Clinician judgement	**Computerised short screening tool**	Clinician judgement	Clinician judgement	Clinician judgement
% and no. detected	20% *n* = 300	**35% n = 525**	20% *n* = 300	20% *n* = 300	20% *n* = 300
Cost for detection (per person)	$5	**$10.00**	$5	$5	$5
Total cost for filter 1	$7500	**$15,000**	$7500	$7500	$7500
*Filter 2: Provider response*					
Provider response (description)	Clinician judgement	Clinician judgement	**Provision of patient distress screening scores and recommendation to clinician**	Clinician judgement	Clinician judgement
% and no. offered treatment	70% *n* = 210	70% *n* = 368	**85% n = 255**	70% *n* = 210	70% *n* = 210
Cost for provider (per person)	$5	$5	**$10**	$5	$5
Total cost for filter 2	$1500	$2625	**$3000**	$1500	$1500
*Filter 3: Patient acceptance*					
Patient acceptance (description)	Patient judgement	Patient judgement	Patient judgement	**Distress scores & recommendation provided to patient**	Patient judgement
% and no. accept treatment	30% *n* = 63	30% *n* = 110	30% *n* = 77	**45% n = 95**	30% *n* = 63
Cost for acceptance (per person)	$0	$0	$0	**$7.50**	$0
Total cost for filter 3	$0	$0	$0	**$1575**	$0
*Filter 4: Treatment efficacy*					
Treatment (description)	Referral to primary care	Referral to primary care	Referral to primary care	Referral to primary care	**Collaborative care model**
Treatment outcome	No longer meets diagnostic criteria for depression	No longer meets diagnostic criteria for depression	No longer meets diagnostic criteria for depression	No longer meets diagnostic criteria for depression	**No longer meets diagnostic criteria for depression**
% and no. treated successfully	30% *n* = 19	30% *n* = 33	30% *n* = 23	30% *n* = 28	**45% n = 28**
Cost for treatment (per person)	$100	$100	$100	$100	**$300**
Total cost for filter 4	$6300	$11,025	$7650	$9450	**$18,900**
*Outcome metrics*					
Total cost	$15,300	$28,650	$18,110	$20,025	$27,900
Cost per patient receiving care	$242.86	$260	$237	$212	$443
Cost per successful outcome	$810	$866	$791	$706	$984
Incremental total cost compared to baseline	n/a	$13,350	$2850	$4725	$12,600
Incremental number of patients successfully treated compared to baseline	n/a	14	4	9	9
ICER	n/a	$942	$704	$500	$1333
Policy Advice	n/a	Compared to usual care this scenario is MORE EXPENSIVE and has BETTER EFFECTIVENESS	Compared to usual care this scenario is MORE EXPENSIVE and has BETTER EFFECTIVENESS	Compared to usual care this scenario is MORE EXPENSIVE and has BETTER EFFECTIVENESS	Compared to usual care this scenario is MORE EXPENSIVE and has BETTER EFFECTIVENESS

Data in bold indicate key changes to the filter input data under the four scenarios

Under the assumptions made for the baseline and four scenarios:

- Compared to baseline, scenario 1 (↑ detection) produced 14 additional incremental successes, scenarios 3 (↑ patient acceptance of treatment) and 4 (↑ treatment effectiveness) produced 9 additional successes, and scenario 2 (↑ provider response) produced 4 additional successfully treated patients;
- Compared to baseline, scenario 3 (↑ patient acceptance) had the lowest cost per additional successful outcome of the four scenarios, and therefore the lowest ICER; Scenario 3 was the most cost-effective of the three non-baseline scenarios;
- Compared to baseline, scenario 4 (↑ treatment effectiveness) had the highest cost per additional successful outcome of the three non-baseline scenarios, and therefore the highest ICER; Scenario 4 was the least cost-effective option of the three non-baseline scenarios;
- Scenarios 2 (↑ provider response) and 1 (↑ detection) had intermediary costs per additional successful outcome and ICER values, compared to baseline.

The model also provides decision makers with information on the total budgetary change required to implement proposed changes to the treatment pathway. Based on the hypothetical data, Scenario 3 (↑ patient acceptance) would require the allocation of an additional $4725 above baseline to deliver an additional 9 successes. Scenario 4 (↑ treatment effectiveness) would require an additional $12,600 to deliver the same number of additional successes. The greatest number of additional successes (*n* = 14) could be achieved under scenario 1 (↑ detection), for a total additional cost of $13,350. The least number of additional successes were achieved (*n* = 4) at a total additional cost of $2850 under scenario 2 (↑ provider response).

## Discussion

Ideally all cancer patients with depression should be identified and treated. However, given increasingly limited healthcare budgets, this simple filter tool can assist decision makers to make transparent decisions about the allocation of scarce resources to best improve depression outcomes in cancer settings. While the simplicity of the tool necessitates some limitations, it should help decision makers to identify and consider relevant parameters that may influence an investment decision. It also helps identify the data that needs to be sourced to help inform decisions, and provides a prompt to utilise the existing research evidence, where available. Use of the model therefore represents a potential improvement on the current situation where there is little or no consideration given to the available evidence.

The filter model is a tool for exploring the impact of changes to the depression treatment pathway on patient outcomes and clinic costs. The results can be used to inform decision makers about the possible returns from investments in a given field. This information provides additional clarity about where resources can or should be allocated for best value for money. In the setting illustrated, the filter model describes, and makes transparent, a logical decision making pathway for considering a range of interventions to improve outcomes for cancer patients experiencing depression. The transparency of this decision making pathway is, in itself, a process to engage and learn from stakeholders, so that these views can be incorporated into the decision making process.

Given the arbitrary nature of the data used to illustrate the filter model, the model results are not designed to make conclusions about which approach to improving treatment of depression is the best or most cost-effective. The model is a theoretical tool which requires empirical testing, and may need to be refined as a result of such testing. Testing of the model across a range of contexts would be helpful, including for example: informing decisions where interventions are potentially very expensive, or where interventions are relatively affordable compared to the alternatives; informing where additional research is critical, such as a dominant parameter with little evidence; and/or educating decision makers regarding the implicit assumptions that are made within alternative options. Despite this, the filter model prompts the user to consider important parameters which impact on depression care, and provides a demonstration of how outcomes might change according to which aspects of depression care are altered. In the absence of readily available evidence, key model parameters can be elicited from content experts, and a range of plausible values can be explored to observe the variability in outcomes. Sensitivity analyses would be recommended where model parameters are varied to their plausible extremes if decisions were to be made from the results of the model.

### Who might use the simple filter model?

The filter model has broad application for treatment centres, health departments, funding agencies and research groups. For treatment centres, the filter model is useful for examining the current care pathway and modelling the consequences of possible changes to this pathway. Under the hypothetical scenarios modelled in this paper, an intervention to increase patient acceptance of treatment by 15% led to the same number of incremental successes as increasing the effectiveness of treatment offered to patients, but at a fraction of the cost. The model can therefore be used to assist in conceptualising the consequences from changes to clinical systems. Through the process of logically considering the consequences from system changes or new interventions, it will be possible to assess the consequential downstream resourcing implications. For example, if the likely impact from a proposed intervention is an increase in the number of cases of depression that will be successfully detected, then the downstream impact would be expected to translate into a rise in the number of patients seeking treatment. Decision makers can then examine existing capacity in the system to plan for the provision of sufficient resources.

For health departments, and within a given field, the model can be used to guide decision making about where to invest limited resources for the best value for money. For example, improving provider response to patients identified as depressed may be more cost-effective than offering detected patients a more effective but more expensive treatment. Users can therefore select the intervention which provides the best outcome within a given budget.

For funding agencies and research groups, the filter model highlights aspects of depression treatment in cancer care where there is a lack of available evidence to help inform decision making. As a consequence, researchers and those who fund them can target their research efforts towards addressing these gaps in the evidence. For example, while considerable research effort has been expended on testing the effectiveness of screening for depression in cancer care [36, 37] and to some extent, for the treatment of depression for cancer patients [38, 39], there is a relative paucity of research examining other barriers to depression care among cancer patients [6], and in particular a lack of intervention research designed to overcome these barriers. There is also an almost complete absence of information available about the costs associated with implementing changes to the depression care pathway in cancer. Researchers and funding bodies urgently need to build in measures of effectiveness and cost effectiveness into future intervention studies.

### Advantages of the model

This model provides a simple and accessible tool for guiding decisions about where to allocate resources to potentially improve depression care in cancer. Key advantages of this model lie in its simplicity and flexibility. While other approaches to modelling such as decision analysis may be more precise, they are also necessarily more complex and resource intensive to undertake [16]. The power of this simple filter model lies in the ability of the model to cope with uncertainty in the input data, to incorporate new research data as it emerges, and to ensure a logical pathway is followed when making decisions about health and medical research and services. The filter model can be easily altered and re-run, allowing a range of assumptions to be modelled to account for variability in input data. The visibility of the key parameters in the model allow scrutiny and the ability to vary these parameters to cope with uncertainty in the input. The model is highly flexible, and could potentially be tailored for use in other settings outside of depression in oncology, including to other outcomes, populations, and interventions. The model also highlights the data needed to make informed decisions on resource allocation and therefore helps to identify gaps in the available data.

### Limitations of the model

The filter model is a simplified tool for guiding the allocation of resources in depression care, and therefore has a number of limitations. The central limitation is that the filter model represents only one of multiple pathways, and does not include indirect or unwanted costs, such as those associated with undetected and untreated depression or the cost of ‘false positives’. Therefore the cost outcomes of the model need to be considered as direct system costs associated with implementing a change from baseline, rather than as overall healthcare system costs. The benefits of any improvement in depression care processes are also likely to be larger than suggested by the model, as broader downstream costs associated with untreated depression will be avoided by any improvement in detection and treatment. These could include, for example, avoided hospitalisations and emergency department visits. These downstream costs are not reflected in the model.

The model also assumes that each filter operates independently, whereas in reality there may be some overlap or interaction between filters. For example, a change in the way that providers respond to the detection of depression (filter 2), or in the type and effectiveness of treatment offered (filter 4), may impact on patient acceptance of the treatment (filter 3). Empirical testing will help to determine whether the static filter approach is an adequate representation of real-world systemic interactions.

Finally, some of the intervention costs may also be better described as costs per provider or per treatment centre, rather than as per patient, as required by the model. For example, costs for an intervention such as electronic screening for depression and provision of provider and patient feedback, could apply across filters and across centres, rather than per patient. In addition, the model assumes that costs are consistent across all patients. In practice there may be some variation in treatment costs, if for example, treatment type or intensity varies according to the patient’s needs or preferences.

## Conclusion

While this simple theoretical filter model needs empirical testing to confirm its functionality (or alternatively to refine and improve the model), it provides a tool to assist decision and policy makers to make transparent decisions about how to best allocate resources to improve depression outcomes in cancer care. These decisions are often made with little or no consideration of the available research evidence [14]. Despite its limitations, the filter model presents an opportunity to improve on current practice by ensuring a logical approach is applied to decision making and that this approach prompts users to consider: i) the relevant available evidence; and ii) the missing evidence that is necessary to make an informed decision. As a consequence of the latter point, the model contributes to identifying gaps in evidence which require more rigorous intervention work to provide reliable data about effectiveness and cost. The authors invite organisations and researchers to implement and test the model and provide suggestions for improvement. A copy of the model is available from the authors on request.
